# Research orientation among general practitioners compared to other specialties

**DOI:** 10.1080/02813432.2021.1880072

**Published:** 2021-02-05

**Authors:** Markku Sumanen, Tiia Reho, Teppo Heikkilä, Pekka Mäntyselkä, Hannu Halila, Kari Mattila

**Affiliations:** a Faculty of Medicine and Health Technology, Tampere University, Tampere, Finland; b Helsinki University Hospital, Helsinki, Finland; c Institute of Public Health and Clinical Nutrition, General Practice, University of Eastern Finland, Kuopio, Finland; d Primary Health Care Unit, Kuopio University Hospital, Kuopio, Finland; e Finnish Medical Association, Helsinki, Finland

**Keywords:** General practice, research work, doctoral degree, primary health care, GP education programmes

## Abstract

**Objective:**

The volume of research work done by general practitioners (GP) is modest compared to other specialties. In order to find out reasons for this we examined the current situation concerning research orientation and factors relating to them among Finnish GPs compared to other specialists.

**Design and setting:**

Data from The Physician 2018 Study were used for our research. The study was undertaken in collaboration with all five medical faculties in Finland and the Finnish Medical Association. It compiled information on physicians` social background, work history and career and research plans as well as their views regarding undergraduate and specialist training, values, and professional identity.

**Subjects:**

The basic study population comprised all Finnish doctors under 70 years of age (*N* = 23,131). Questionnaires were sent to doctors born on even-numbered days (*n* = 11,336). Altogether 5,214 (45.8%) responded. Responses from GPs (*n* = 796) were compared with those of doctors in other specialties (*n* = 3,514).

**Main outcome measures and results:**

The respondents were asked about their current intention to undertake a doctoral degree. Factors associated with this were analysed. Only 7.3% of GPs had completed a doctoral degree. The corresponding figure in other specialties was 32.3% (*p* < 0.001). In general practice the current intention to undertake a doctoral degree had only slightly increased over ten years. Most GPs had also decided not to undertake a doctorate. The main factors associated with the current intention to complete a doctoral degree were interest in attaining a senior position (OR 3.43, 95% CI 2.25–5.24), a position in a university hospital district (OR 2.89, 95% CI 1.69–4.94) or other sector than primary care (OR 1.87, 95% CI 1.18–2.96), one’s father being a doctor (OR 2.01, 95% CI 1.09–3.72) and male gender (OR 1.63, 95% CI 1.05–2.54).

**Conclusion:**

Research work in primary health care has been quite sparse. In general practice there is a need to increase teaching and guidance in research work.

**Key points**
- Research work in primary health care is not very common.
- Only 7.3% of GPs had completed their doctorate compared to 32.3% in other specialties.
- A main factor associated with the current intention to complete a doctoral degree was interest in attaining a senior position.

## Introduction

Undertaking research work and thereby developing medical knowledge and improving patients’ health has been considered an essential part of the physician’s job. In Finland, there are over one hundred evidence-based Current Care Guidelines written by the best specialists in their field [1]. General practitioners (GPs) are involved in writing and updating most of these guidelines. Nevertheless, research work in primary health care is not very widespread. According to a Finnish study in the year 2013, only ten per cent of GPs had undertaken or completed a doctoral degree, while the corresponding proportion among all doctors was 34% [2]. However, reasons for the low proportion have not been examined.

Internationally, primary health care professionals have not been very active in performing research work [3]. There may also be negative attitudes towards research. The gap between theoretical research and practical work of GPs and the domination of research by specialists have been found important reasons for skepticism about research [4]. According to a previous study, most Finnish GPs thought that the amount of training in research had been insufficient during their specialization process [5]. However, in recent years in the Nordic countries, there have been aspirations to enhance research work in primary health care [6,7]. For example, primary care research courses and scientific congresses and seminars have been organized in Finland [8]. Research networks have also been established [9]. Moreover, participation in a scientific congress of general practice is currently required during the specialization process.

General practice is the largest specialty in Finland [10]. Unfortunately, its contribution to research work and number of published articles is rather modest compared to other major specialties. Only a small proportion of the authors of peer-reviewed studies in primary health care have worked in community health centres [11]. This article examines the current situation concerning doctoral studies and factors relating to them among general practitioners. Moreover, it considers how the current intention to complete a doctorate changed between 2008 and 2018.

## Material and methods

**Figure 1.**
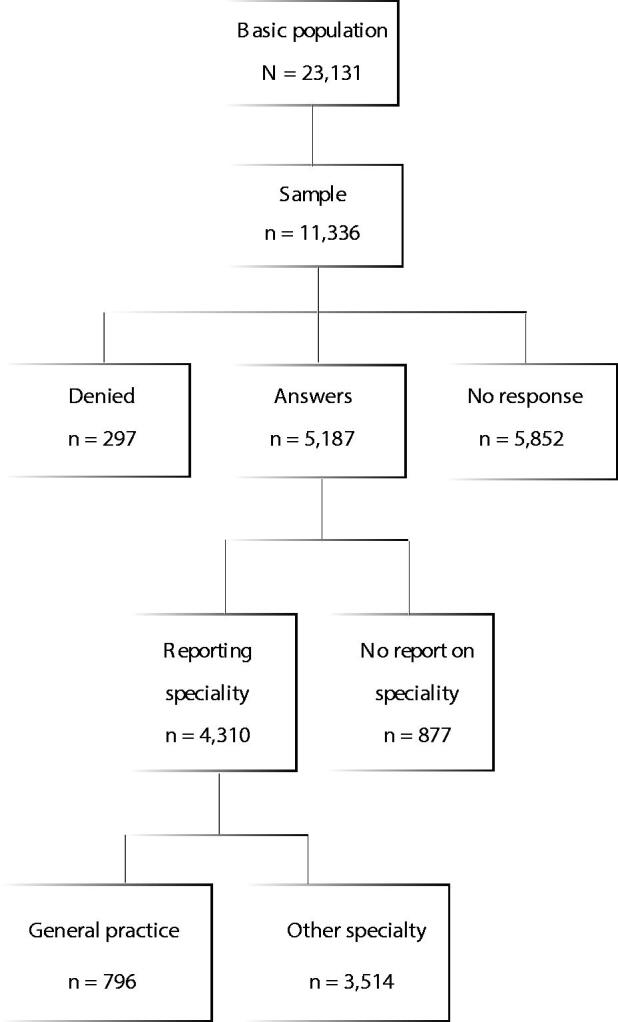
Flow chart of the Finnish Study 2018 population.

The Physician 2018 Study was undertaken in collaboration with all the five medical faculties in Finland and the Finnish Medical Association. It followed previous studies conducted in 1988, 1993, 1998, 2003, 2008, and 2013. The study compiled information on the social background, work history, labour market position, and the career and research plans of physicians working in the medical profession [12]. Moreover, doctors were asked about their views regarding undergraduate and specialist training, values and professional identity. In order to improve comparability, most of the questions were formed already in the first study in 1988, but new questions have been added in later questionnaires. In 2013 and 2018, both postal and electronic questionnaires were used. Addresses were collected from the database of the Finnish Medical Association, which maintains the details of doctors licensed in Finland. Those who had declined to give their personal information were excluded. The basic study population in the Physician 2018 Study comprised all Finnish doctors under 70 years of age (*N* = 23,131). In previous questionnaires, doctors born on either even- or odd-numbered days were included in the study. This time doctors born on even-numbered days were drawn from this basic study population (*n* = 11,336). The formation of the data is presented in Figure 1.

Altogether 5,187 doctors responded to the questionnaire, a response rate of 45.8%. Women responded more actively than men: the corresponding response rates were 49% and 39%. The response rate varied by age group, being the lowest (40%) in the 35- to 44-year-old age group and highest (55%) in the oldest age group (65‒69 years). Medical specialists (50%) answered more often than unspecialized doctors (38%). However, we do not know the response rates among separate specialties. [13] Altogether 877 respondents did not report their specialty. The analysed population thus comprised 4310 doctors.

The respondents were asked their gender, marital status, the spouse/mother/father being a doctor, having children, current senior position as well as interest in attaining a senior position, working sector (primary care or other), working at a university hospital district, and the population of the working municipality.

Doctors in primary health care were compared with other specialties using crosstabulations. Gender, age, and factors related to research interests and education were compared. The proportion of women was greater among those in general practice compared to other specialties. Doctors in general practice were also younger than doctors in other specialties (Table 1).

**Table 1.** Respondents’ distributions (%) and comparisons of sociodemographic features and opinions to education in research during education among doctors in general practice and other specialties.

	General practice *N* = 784		Other specialties *N* = 3446		*p* Value
	N	%	n	%	
Gender^a^					<0.001
Women	580	74.0	2151	62.4	
Men	204	26.0	1295	37.6	
Age groups^a^					<0.001
<35	157	20.1	480	14.0	
35‒44	206	26.4	764	22.3	
45‒54	135	17.3	836	24.4	
55‒64	190	24.4	927	27.1	
65‒69	92	11.8	413	12.1	
Interest in research work had some influence on studying medicine	158	20.2	1110	32.4	<0.001
Too little research education during medical studies^b^	129	39.7	501	49.5	0.002
Opportunities to do research work had some influence on choosing specialty	81	10.4	1375	39.7	<0.001
Too little research education during specialization process	473	62.1	1591	46.8	<0.001

Organization of the alternatives for answers: far too little and too little combined, as well as appropriate amount, too much and far too much combined. Crosstabulation and chi-squared test were used in the analyses.

^a^Not every respondent reported their age and gender (*n* = 12 in general practice and *n* = 68 in other specialties).

^b^Most respondents did not answer this question.

The respondents were asked their specialty and whether they continued to specialize in it. Moreover, they were asked about their current intention in terms of doctoral studies. The answer options were: 1. I have not made a decision on doctoral studies; 2. I have decided not to undertake a doctorate; 3. I am going to undertake a doctorate, but I am not sure of the topic; 4. I am going to undertake a doctorate and I have already chosen the topic; 5. The doctorate is ongoing; and 6. The doctorate has been completed. The answers were analysed according to age, gender, family matters, workplace, working sector and working position (chief physician or not). The same questions were also asked in the 2008 and 2013 studies [14,15].

Opinions on research work were asked using the following questions: ‘To what extent did interest in research work influence you to study medicine?’ ‘To what extent did you receive education and guidance in research work during your medical studies?’ ‘To what extent did having opportunities to do research work influence you when choosing a specialty?’ ‘To what extent did you receive education and guidance in research work during your specialization process?’ The options for these questions were far too little, too little, appropriate amount, too much, and far too much. In the analyses, the first two and the last three options were combined. Moreover, the proportion of GPs currently intending to undertake a doctoral degree was compared with the figure from 2008. The respondents were also asked whether they conducted research work other than that for doctoral studies.

Finally, univariate and multivariate logistic regression analyses were used to investigate factors associated with the GPs current intention to undertake a doctorate or current situation of doctoral studies, the dependent variable being the intention. The analysed factors were gender, marital status, the spouse/mother/father being a doctor, having children, current senior position as well as interest in attaining a senior position, working sector (primary care or other), working at a university hospital district, and the population of the working municipality. It should be noted that Finland is divided into twenty hospital districts, of which five are university districts with a medical faculty and a university hospital. In these analyses, no decision on doctoral studies and the decision not to undertake a doctorate were combined. The current intention to undertake a doctorate, current doctoral studies, and completed doctoral studies were also combined.

In the analyses, those who reported general practice as their specialty were compared to those with other specialties. The data were analysed using SPSS version 23.0. The results are presented as frequencies, percentages, and odds ratios (OR) with 95% confidence intervals (CI).

The Research Ethics Committee of Kuopio University Hospital has confirmed that according to Finnish legislation, no ethical approval was needed for the study. The respondents also gave their written permission to publish the results of the study.

## Results

In total 4,230 doctors reported their specialty, of which 796 respondents (women 74%) were GPs, 528 of them specialists in general practice.

Interest in research work had less influence on studying medicine among GPs compared to those in other specialties. In addition, opportunities to do research work had less influence on choosing the specialty among GPs than among those in other specialties. Compared to those in other specialties, GPs less often considered themselves having had too little research education during medical studies. However, GPs more often considered themselves as having had too little research education during specialization compared to other specialists (Table 1).

Of those reporting general practice as their specialty, 7.3% (*n* = 58) had already completed a doctorate, the proportions being 11.3% among men (*n* = 23) and 5.9% among women (*n* = 34), *p* = 0.011. In primary health care centres 10.9% (*n* = 6) of chief physicians had completed a doctorate, the corresponding proportion being 4.7% (*n* = 18) among other doctors (*p* = 0.060) in general practice.

**Figure 2.**
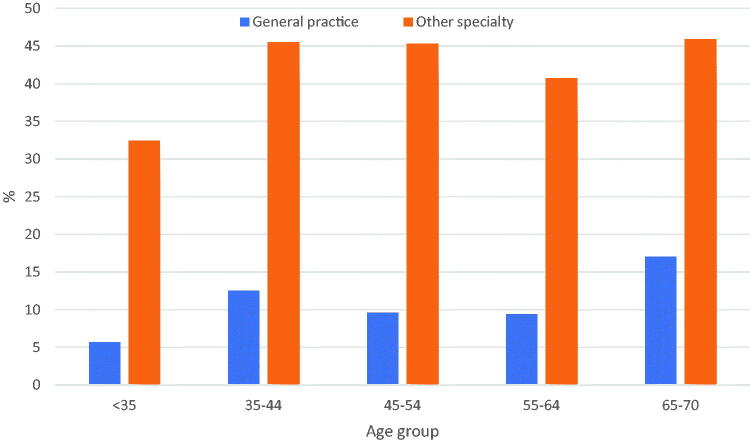
Proportion (%) of GPs and doctors in other specialties in different age groups having started or completed a doctorate.

Compared to other specialties, doctoral studies were less common among general practitioners (Table 2). Half of them had decided not to undertake a doctorate, and one third had not decided. Seven per cent had already completed a doctorate, while in other specialties the corresponding proportion was 32%. Current or completed doctoral studies were less common among GPs in all age groups. General practitioners also defended their doctoral dissertation at a later age compared to other specialists (Figure 2).

**Table 2.** Intention on completing a doctorate in the Physician 2018 Study.

	General practice *N* = 793		Other specialties *N* = 3,496	
	n	%	n	%
No decision on doctoral studies	284	35.8	718	20.5
Decided not to complete a doctorate	403	50.8	1,084	31.0
Going to undertake a doctorate, but not sure about the topic	15	1.9	130	3.7
Going to undertake a doctorate, already chosen the topic	8	1.0	81	2.3
The doctorate is ongoing	25	3.2	354	10.1
The doctorate has been completed	58	7.3	1,129	32.3

Proportion (%) of answers among general practice and other specialties. Crosstabulation and chi-squared test were used in the analyses.

*p* < 0.001.

The rates of current intention to undertake a doctorate had not changed significantly over ten years (2008–2018), as only a slight increase in intention was found (from 12.2% to 13.4%). The proportion of those who had decided not to undertake doctoral studies had declined from 53.6% to 50.8%.

Academic research work other than that for the purposes of a doctorate was also minor in general practice. Only 6.0% reported doing such research. The corresponding figure was 30.5% among other specialties (*p* < 0.001).

In the univariate logistic regression analysis, the current intention of GPs to undertake doctoral studies associated with an interest in attaining a senior position (OR 3.43, 95% CI 2.25‒5.24), a workplace at a university district (OR 2.89, 95% CI 1.69‒4.94), and a workplace other than in primary care (OR 1.87, 95% CI 1.18‒2.96), as well as with one’s father being a doctor (OR 2.01, 95% CI 1.09‒3.72) and being of the male gender (OR 1.63, 95% CI 1.05‒2.54). Marital status, the mother or spouse being a doctor, having children, holding a senior position, and the population of the working municipality were not associated with the current intention. In the multivariate regression analysis, interest in attaining a senior position (OR 5.23, 95% CI 2.87‒9.54) and a workplace at a university hospital district (OR 3.36, 95% CI 1.79‒6.31) remained statistically significant for GPs undertaking doctoral studies (Table 3).

**Table 3.** Factors associated with intention to undertake a doctorate among GPs in univariate and multivariate logistic regression analyses.

	Univariate model	Multivariate model^a^
	OR (95% CI)	OR (95% CI)
Gender		
Female (n = 580)	1	1
Male (n = 204)	**1.63 (1.05–2.54)**	1.81 (0.99–3.29)
Marital status		
Single (n = 68)	1	1
In a relationship (n = 713)	1.62 (0.68–3.84)	1.00 (0.00–>10)
Spouse is a doctor		
No (n = 567)	1	1
Yes (n = 146)	1.03 (0.61–1.75)	1.09 (0.57–2.09)
Mother is a doctor		
No (n = 728)	1	1
Yes (n = 48)	1.15 (0.50–2.63)	1.34 (0.47–3.85)
Father is a doctor		
No (n = 707)	1	1
Yes (n = 68)	**2.01 (1.09–3.72)**	2.20 (0.95–5.10)
Children		
None or only one (n = 174)	1	1
At least two (n = 554)	0.80 (0.49–1.29)	0.63 (0.35–1.16)
Being in a senior position		
No (n = 644)	1	1
Yes (n = 145)	1.37 (0.84–2.25)	0.62 (0.31–1.22)
Interested in attaining a senior position		
Little (n = 532)	1	1
Much (n = 254)	**3.43 (2.25–5.24)**	**5.23 (2.87–9.54)**
Working sector		
Primary care (n = 536)	1	1
Other (n = 168)	**1.87 (1.18–2.96)**	1.58 (0.87–2.87)
Population of working municipality		
< 50.000 (n = 307)	1	1
≥ 50.000 (n = 393)	1.41 (0.91–2.19)	0.99 (0.57–1.73)
Working at university hospital district		
No (n = 259)	1	1
Yes (n = 440)	**2.89 (1.69–4.94)**	**3.36 (1.79–6.31)**

ORs with 95% CI.

^a^All analysed factors are in the multivariate model.

Statistically significant associations are in bold.

## Discussion

The main finding of our study was that undertaking a doctorate was less common among general practitioners compared to other specialties. We also found that the current intention to undertake a doctoral degree in general practice had only slightly increased over ten years. Most GPs had also decided not to undertake a doctorate. It also seems that already during medical studies, they were not planning to do research work in the future.

The strength of our study is that the Finnish Physician Study has provided data on Finnish physicians every five years for the past 30 years. Moreover, the data are large, and may be considered representative of the whole population of physicians in Finland. In addition, the proportion of GPs answering the questionnaires is also about the same as the proportion of GPs in Finland. The response rate has declined, but it is still quite appropriate by international comparison [16]. Any non-response analysis has not been performed.

One weakness of the study may be that not all respondents had yet completed their specialization. Therefore, some participants may not have answered questions concerning specialist training. However, it is probable that those still in specialization have answered the questionnaire as actively as those already having specialized. In addition, not all respondents answered every question. However, this is probably true of both those in general practice and those in other specialties. Another weakness may be that there is a possibility for overcontrolling in the multivariate analysis, as we have put all analysed variables into the model.

There are several challenges and barriers to research work in primary health care. General practice has lacked a research tradition [17]. Therefore, many GPs are not familiar with research, and this kind of activity may be considered additional and stressful. It might also be the case that GPs are busy with their work, with no extra time for research. Many GPs also feel exhausted due to their workload. Another problem is funding, which in primary health care often is quite difficult to obtain. It is known that medical PhD students have in general the lowest income of all doctors in the Nordic countries. Thus, GPs lack incentives to start doctoral studies, as they will lose money by undertaking a PhD [18].

In our study, chief physicians in primary health care centres had undertaken a doctorate more often compared to other doctors. Interest in other activities than the regular physician’s practice may have led to both interest in academic work and to leading positions. Academic merits may also increase the chief physicians’ valuation, as well as the examination skills required of managers. Chief physicians are usually older than other doctors. They have thus had more time to gain an education in research and undertake a doctorate. In hospital specialties, academic merits are important in attaining a senior position, and in university hospitals they are absolutely required. The situation is different in health care centres, where administrative skills are considered more important. In a Canadian study, willingness to undertake leadership was mostly associated with leadership experiences and perceptions of mentorship, while academic activities for professional development were less important [19].

One possible difference between GPs and hospital doctors concerning research activity may be that research is a part of daily discussions in hospitals. Our finding that GPs in University Hospital districts were more often engaged in research may be that they were closer to the research environment at the university. Therefore, they may find it easier to build research networks for GPs.

In our study, one in three GPs had not yet decided whether to undertake a doctorate in the future. This population can thus be regarded as the group upon which to focus special efforts, and there are opportunities to do so. Research courses for GPs have been organized, and this activity will be further improved. A research agenda for general practice was published at the WONCA Europe Conference in 2009 [20]. This agenda was set by the European General Practice Research Network (EPGRN) organization, which has enhanced research work in primary health care for many years [21]. More research networks in primary health care services would also be useful [22]. This kind of research network has been successful in Scotland [23]. The volume of research dealing with or being conducted within primary care is somewhat smaller in Finland than in the other Nordic countries, the Netherlands or Great Britain [11]. Completing a doctorate is only part of scientific research education. The education also includes getting acquainted in scientific literature, organizing methodological and theoretical courses, writing original and review articles as well as participating national and international congresses.

Although research activity in primary health care is minor, many chief physicians in Finland have concluded that research belongs in health care centres [24]. Moreover, the supervision of research is hardly a problem. The departments of general practice at universities are continuously seeking new doctoral candidates. These departments have their own research data, and linkages to national health records are often available [25]. In addition, the accumulation of electronic information systems may facilitate the collection of research data [26]. The development in information technology has made it possible to participate in research collaboration regardless of place of residence or work.

## Implications

Thus far, research work among general practitioners has been quite small. There is a need to increase teaching and guidance in research work. General practice has many topics worthy of research, and unselected patient materials provide numerous opportunities. The departments of general practice in medical faculties are at the front-line enhancing research activity. There are still many doctors in primary health care who have not made their final decision regarding research work. Undertaking research work will help to strengthen the role of GPs in the health care system and hopefully improve the state of health of primary care patients and citizens in general.
